# Effects of high‐intensity intermittent exercise versus moderate‐intensity continuous exercise on renal hemodynamics assessed by ultrasound echo

**DOI:** 10.14814/phy2.15925

**Published:** 2024-01-23

**Authors:** Shotaro Kawakami, Tetsuhiko Yasuno, Saki Kawakami, Ai Ito, Kanta Fujimi, Takuro Matsuda, Shihoko Nakashima, Kosuke Masutani, Yoshinari Uehara, Yasuki Higaki, Ryoma Michishita

**Affiliations:** ^1^ Graduate School of Sports and Health Science Fukuoka University Fukuoka Japan; ^2^ Faculty of Sports and Health Science Fukuoka University Fukuoka Japan; ^3^ The Fukuoka University Institute for Physical Activity Fukuoka Japan; ^4^ Division of Nephrology and Rheumatology, Department of Internal Medicine Fukuoka University School of Medicine Fukuoka Japan; ^5^ Department of Rehabilitation Fukuoka University Hospital Fukuoka Japan

**Keywords:** AKI biomarker, high‐intensity intermittent exercise, moderate‐intensity continuous exercise, renal hemodynamic

## Abstract

High‐intensity intermittent exercise (HIIE) has become attractive for presenting a variety of exercise conditions. However, the effects of HIIE on renal function and hemodynamics remain unclear. This study aimed to compare the effects of HIIE and moderate‐intensity continuous exercise (MICE) on renal hemodynamics, renal function, and kidney injury biomarkers. Ten adult males participated in this study. We allowed the participants to perform HIIE or MICE to consider the impact of exercise on renal hemodynamics under both conditions. Renal hemodynamic assessment and blood sampling were conducted before the exercise (pre) and immediately (post 0), 30 min (post 30), and 60 min (post 60) after the exercise. Urine sampling was conducted in the pre, post 0, and post 60 phases. There was no condition‐by‐time interaction (*p* = 0.614), condition (*p* = 0.422), or time effect (*p* = 0.114) regarding renal blood flow. Creatinine‐corrected urinary neutrophil gelatinase‐associated lipocalin concentrations increased at post 60 (*p* = 0.017), but none exceeded the cut‐off values for defining kidney injury. Moreover, there were no significant changes in other kidney injury biomarkers at any point. These findings suggest that high‐intensity exercise can be performed without decreased RBF or increased kidney injury risk when conducted intermittently for short periods.

## Graphical abstract

Visual summary of the current study.

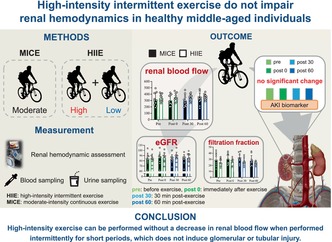

## 1 INTRODUCTION

It is generally known that habitual exercise is effective in providing health benefits, including the improvement of cardiorespiratory fitness and vascular function and antihypertensive effects (Rowe et al., 2014; Watson et al., 2018). In patients with chronic kidney disease (CKD), previous studies have reported that increased physical activity suppresses renal function decline (Beetham et al., 2022; Michishita et al., 2016a, 2016b, 2017), and habitual exercise may have renoprotective effects in animals (Avin et al., 2019; Hu et al., 2020; Kosaki et al., 2019) and humans (Greenwood et al., 2015; Kosaki et al., 2018; Santana et al., 2017). Habitual exercise may play an important role as a preventive activity against renal function decline. However, safe and effective exercise conditions (intensity and duration) for patients with CKD, with and without dialysis, have not been inadequately discussed. One of the reasons for this is that exercising decreases renal blood flow (RBF) in an exercise intensity‐dependent manner through the redistribution of blood flow (Kawakami et al., 2018; Kotoku et al., 2019). Because renal hemodynamics are strongly influenced by exercise intensity and duration, decisions regarding exercise intensity and duration are particularly important. Our group recently investigated the relationship between exercise intensity and duration with renal hemodynamics (Kawakami et al., 2022). Our findings demonstrated that moderate‐intensity continuous exercise (MICE) maintains RBF and does not impair renal function, suggesting that MICE may be a safe and effective exercise condition for patients with CKD. Among the wide range of variations in exercise patterns for clinical practice, MICE has been proven to have beneficial systemic effects, including the reduction of morbidity and/or mortality, and is usually recommended (Liguori et al., 2021).

Patients with CKD are susceptible to low cardiorespiratory fitness and muscle wasting due to poor nutritional status and physical inactivity. There is a growing interest in high‐intensity intermittent exercise (HIIE) as an exercise condition that can be effective in a short period. Moreover, the efficacy and safety of HIIE for healthy populations and patients with cardiovascular disease (CVD) have been demonstrated (Ramos et al., 2015; Taylor et al., 2020; Weston et al., 2014). Beetham et al. recently reported that HIIE might be a feasible and safe exercise method for improving exercise capacity in patients with CKD (Beetham et al., 2019). Thus, the advantages of HIIE are that it allows for high‐intensity exercise in a short period and is beneficial for improving exercise capacity and skeletal muscle mass or strength with efficiency. HIIE is quite attractive for presenting a variety of exercise conditions. However, the effects of HIIE on renal function and hemodynamics remain unclear.

Several studies have shown that novel acute kidney injury (AKI) biomarkers are increased following exercise (Bongers et al., 2018; Junglee et al., 2012, 2013), the changes of these biomarkers are dependent on the duration and intensity of exercise. Most of studies investigated changes in AKI biomarkers following high‐intensity endurance exercise such as marathon running (Mansour et al., 2017; McCullough et al., 2011), to our knowledge, only one study has examined the effect of HIIE on AKI biomarkers. Juett et al. (Juett et al., 2021) found that HIIE increases AKI biomarkers regardless of hydration status and suggested that kidney injury due to HIIE is mediated by a reduction in RBF; however, RBF was not directly measured in that study. Thus, how HIIE influences renal hemodynamics remains unknown. Therefore, it is clinically important to elucidate the effect of HIIE on renal hemodynamics, renal function, and AKI biomarkers to develop means for a feasible and safe exercise condition to prevent renal function decline.

We evaluated changes in renal hemodynamics following exercise using ultrasound echo in previous studies (Kawakami et al., 2018, 2022; Kotoku et al., 2019). Renal hemodynamic assessment using ultrasound echo is non‐invasive, can rapidly visualize blood vessels, and allows for repeated measurements over time that are less stressful to participants than the traditional methods. Furthermore, renal hemodynamic assessment using ultrasound echo enables the evaluation of not only RBF but also other renal hemodynamic parameters. We therefore used ultrasound echo to determine detailed renal hemodynamic responses, including RBF, to HIIE and MICE.

The acute effects of HIIE on peripheral vascular function recently have been reported (Shi et al., 2022; Weston et al., 2022). Recent research has found that acute HIIE performed with interval improved brachial artery flow‐mediated dilation (FMD) in healthy adults (Weston et al., 2022). There were wall shear stress (WSS) and reactive hyperemia index (RHI) after HIIE were significantly higher than that at baseline, suggesting HIIE could significantly improve WSS and vascular endothelial function (Shi et al., 2022). Furthermore, a single bout of HIIE elicits greater shear rate in the internal carotid artery compared with a matched bout of MICE (Ogoh et al., 2021). Considering increases in peripheral shear stress are greater with higher‐intensity exercise (Green et al., 2002) and shear stress appears to be the primary mechanism underlying acute exercise‐induced improvements in peripheral vascular function (Tinken et al., 2009), HIIE may be more beneficial for peripheral vascular function. Therefore, we hypothesized that HIIE may not have an adverse effect on renal hemodynamics and function, and AKI biomarkers in middle‐aged individuals. Hence, this study aimed to compare the effects of HIIE and MICE on renal hemodynamics, including RBF, using ultrasound echo in middle‐aged individuals. Additionally, we examined changes in renal function parameters and AKI biomarkers following HIIE.

## 2 MATERIALS AND METHODS

### 2.1 Ethical approval

All procedures involving human participants were performed in accordance with the ethical standards of the institutional and/or national research committee at which the studies were conducted (Ethics Committee of Fukuoka University Approval No. 22‐06‐M1) and with the 1964 Helsinki Declaration and its later amendments or comparable ethical standards. All potential risks and procedures were explained to the participants, who gave their written informed consent to participate in this study.

### 2.2 Participants

Ten adult males (age: 37 ± 8 years; height: 1.72 ± 0.05 m; weight: 67.4 ± 6.0 kg) participated in this study. The inclusion criteria were as follows: (1) persons with relatively preserved renal function (eGFR ≥60 mL/min/1.73 m^2^), (2) adult male over 18 years old without serious or progressive disorders and symptoms, (3) persons who do not suffer from specific underlying diseases (cardiovascular or cerebrovascular disease, receiving dialysis) or a history of associated symptoms, (4) persons taking no medications that affect systemic hemodynamics (e.g., β‐blockers, calcium antagonists, renin‐angiotensin‐system inhibitors), (5) persons who have been fully informed of the purpose and content of this study and who have given their consent in writing of their own free will based on a thorough understanding of the purpose and content of the research.

At the time of designing the present study, to our knowledge, there were no published data to inform the effect size of manipulating HIIE on renal hemodynamics. Therefore, the sample size was calculated using G × power version 3.1.9 software (Düsseldorf University, Düsseldorf, Germany) considering the acute effects of HIIE on cerebral hemodynamics based on a previous study (Ogoh et al., 2021) to generate a power of 80% and an alpha risk of 5%. A sample size of 12 participants was considered statistically appreciate. Before participation, they were subjected to a medical checkup, including electrocardiography, to ensure that they were fit for this study. 12 healthy middle‐aged adults were recruited in this study. However, one participant did not complete the experiment due to an unrelated injury, and one participant unable to complete the experiment due to participant availability. Consequently, data are presented as *n* = 10 throughout the experiment. The participants avoided strenuous exercise the day before testing and fasted 8 h prior to testing (drinking water was acceptable). They avoided breakfast and caffeine before carrying out the exercise on the day.

### 2.3 Incremental exercise tests for the determination of the optimal exercise intensity

All participants performed an incremental exercise test using a cycle ergometer (Lode, Corival, Netherlands) to determine the optimal exercise intensity, as described previously (Kawakami et al., 2018). All the incremental exercise tests were conducted according to the termination criteria for clinical exercise testing established by the American College of Sports Medicine (Liguori et al., 2021). The criteria were as follows: (1) leveling off of oxygen intake; (2) blood lactate levels >8 mmol/L after exercise; (3) leveling off of heart rate (HR); (4) rate of perceived exertion (RPE) >18; and (5) respiratory exchange ratio >1.15.

During the incremental exercise test, HR, blood pressure, and RPE were consistently measured. Respiratory gas was measured using the mixing chamber method with mass spectrometry equipment for biogas analysis (ARCO‐2000 MET; Arco System, Kashiwa, Japan). Peak oxygen uptake (VO_2peak_) was calculated using the data obtained during the incremental exercise test. Blood was drawn from the ear lobe at rest and 1‐min intervals during exercise to determine blood lactate concentrations. In this study, we determined the intensity of the lactate threshold (LT) and the onset of blood accumulation (OBLA) from the results of the incremental exercise test. Five technicians assessed the first steep‐increase point of blood lactate as LT and the second steep‐increase point as OBLA (equivalent to a blood lactate level of 4 mmol/L) from visual inspection of graphical plots of LA versus workload. The LT and OBLA intensities were used as indicators of moderate and high intensities based on our previous findings (Kawakami et al., 2018; Tobina et al., 2006). The LT and OBLA were obtained from the means of three out of five values, excluding the maximum and minimum values obtained from the participants.

### 2.4 The consideration of the impacts of HIIE and MICE on renal hemodynamics and the kidneys

**FIGURE 1:**
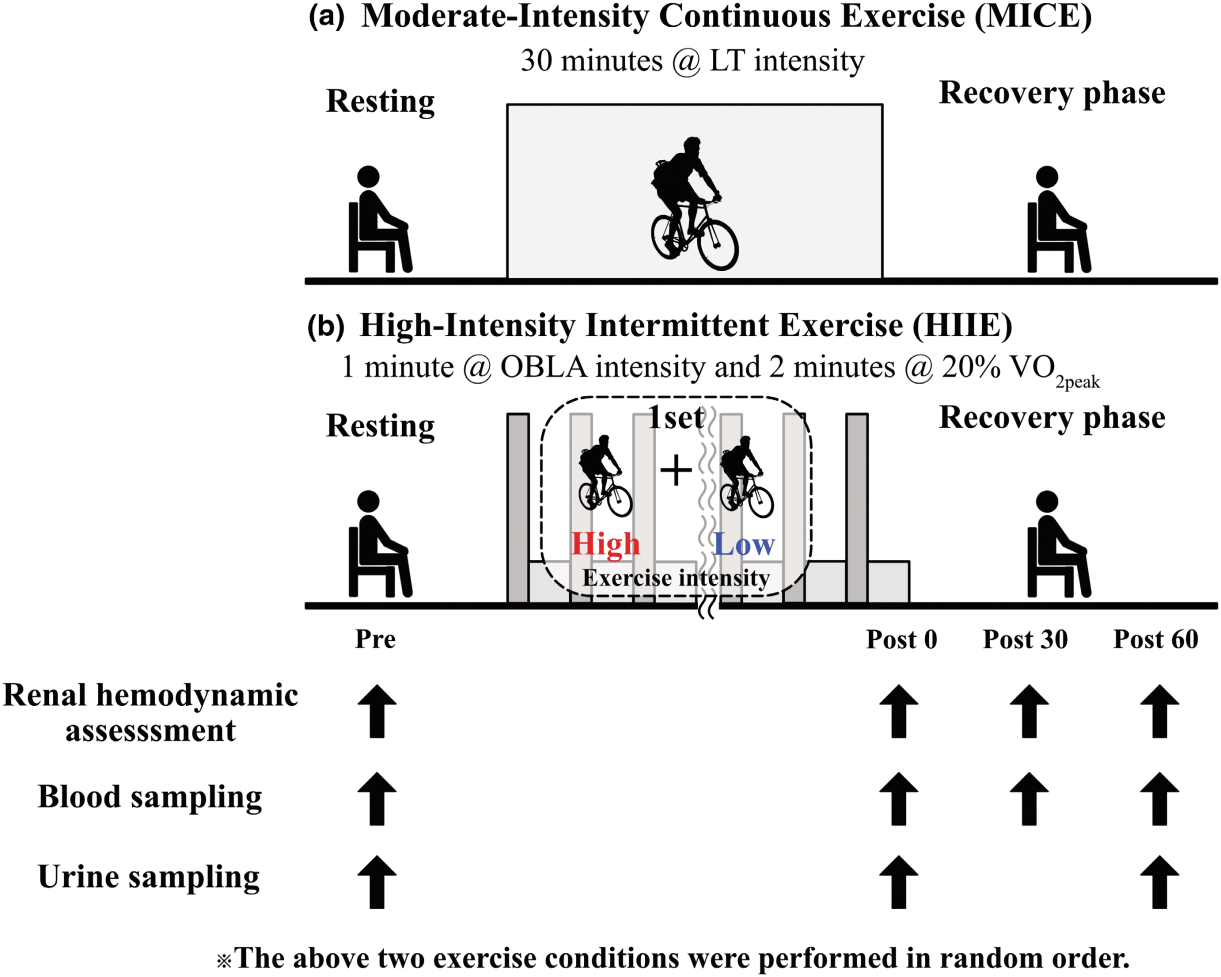
Experimental protocol. All participants performed (a) MICE (30 min at LT intensity) or (b) HIIE (1 min at OBLA intensity and 2 min at 20% VO_2peak_) using cycle ergometer. The number of exercise sessions of intermittent exercise at OBLA intensity was adjusted so that the total energy consumption of both exercise conditions was the same. The above two exercise conditions were performed in random order, with at least 1 week as a washout period between the two conditions. Renal hemodynamics assessment and blood sampling were performed before exercise (*Pre*), immediately after exercise (*Post 0*), 30 min (*Post 30*) and 60 min (*Post 60*) after exercise. Urine samples were taken in *Pre*, *Post 0*, and *Post 60*.

Upon arrival at the laboratory, each participants urinated, drank water, and rested for 5 min. Second, renal hemodynamics were assessed using ultrasound echo. Blood samples were collected to determine the blood biochemistry following renal hemodynamics assessment. Each participant performed MICE (30‐min at LT intensity) or HIIE (1 min at OBLA intensity and 2 min at 20% VO_2peak_) on a cycle ergometer in a random order, with at least 1 week washout period between the two conditions (Figure 1). The number of exercise sessions of HIIE was adjusted so that the total energy consumption of both exercise conditions was the same. To match the total energy consumption of HIIE and MICE conditions, calculating the energy expenditure in both conditions using the following equation (Liguori et al., 2021): Energy expenditure (kcal) = [[[3.5 + 3.5 + [1.8 × workload (watts) × 6.12]/body weight (kg)]/3.5]–1] × exercise duration (h) × body weight (kg) × 1.05.

Renal hemodynamics assessment and blood sampling were performed before exercise (*Pre*), immediately after exercise (*Post 0*), and 30 min (*Post 30*), and 60 min (*Post 60*) after exercise. Urine samples were collected *Pre*, *Post 0*, and *Post 60*. HR and RPE were consistently measured throughout experiment and blood pressure was measured *Pre*, *Post 0*. We evaluated the influence of different combinations of intensity and duration on renal hemodynamics during exercise using ultrasound echo. In addition, blood and urine samples were obtained before and after exercise, in recovery period to explore the mechanisms responsible for renal hemodynamic regulation and the degree of strain on the kidneys. The participants received free hydration during and after the experiment.

### 2.5 The assessment of renal hemodynamics

**FIGURE 2:**
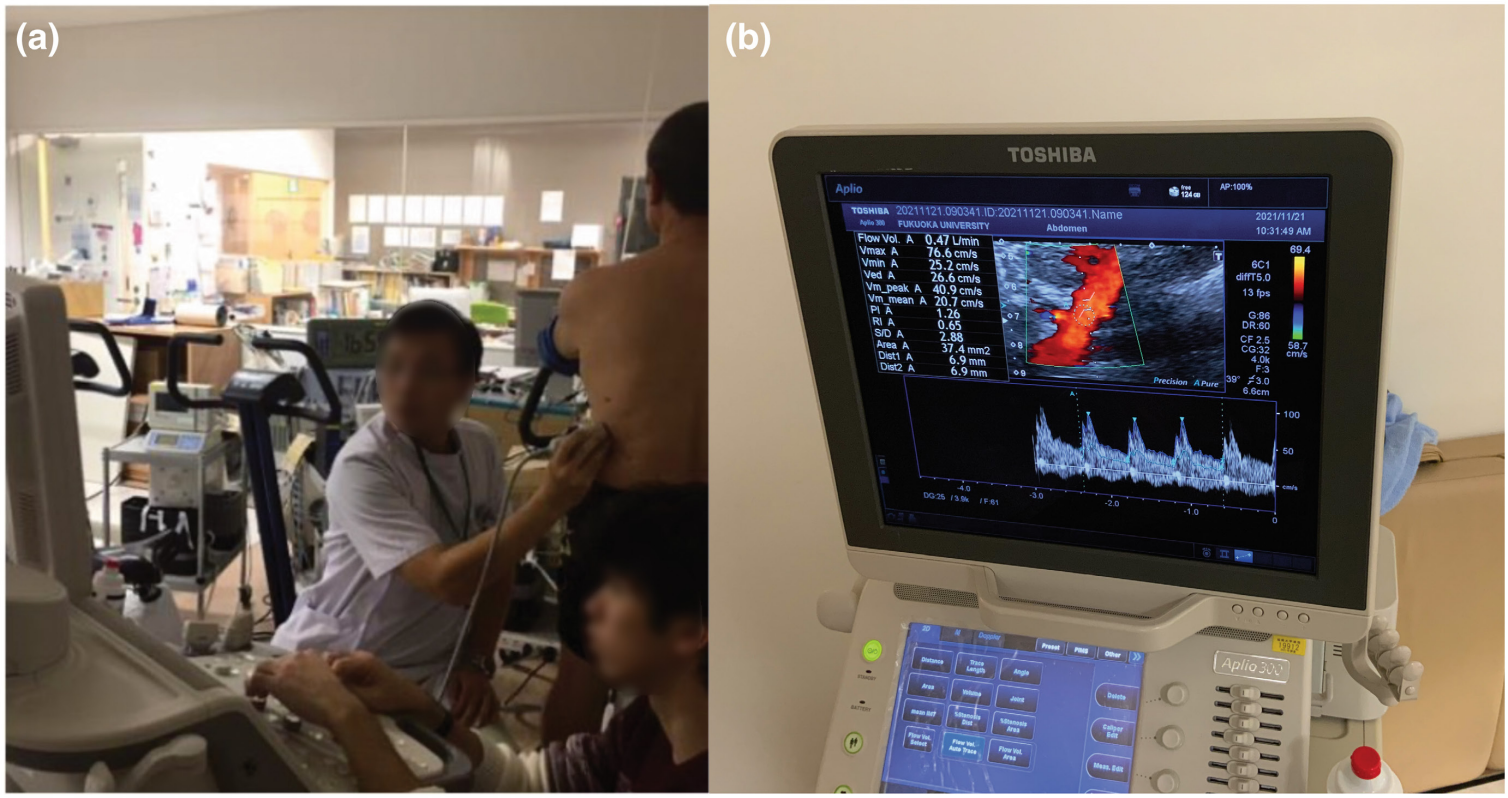
Experimental set‐up and Doppler ultrasound imaging of the kidney. (a) measuring scene, the technician placed the probe on the left lumbar region of the participant in a seated position. (b) The renal artery is drawn, and the technician moved the cursor to the renal artery and measured blood flow velocity (BFV) using the pulse Doppler method, with the average of three Doppler waveforms defined as the mean blood flow waveform. Cross‐sectional area (CSA) is then determined, and renal blood flow (RBF) is calculated based on BFV and CSA using the following equation: RBF (mL/min) = BFV (cm/s) × CSA (mm^2^) × 60 (s). The value for Flow Vol (i.e., renal blood flow), Vmax (i.e., peak systolic flow velocity), Ved (i.e., end‐diastolic flow velocity), Vm_mean (i.e., blood flow velocity), renal pulsatility index (rPI), renal resistive index (rRI), Area (i.e., cross‐sectional area), and Dist (i.e., diameter) are presented.

Renal hemodynamics were assessed using the pulse Doppler method with a 3.5 MHz convex electronic scanning probe of an ultrasound system (Aplio 300; Toshiba Medical Systems) as described previously (Figure 2) (Kawakami et al., 2018, 2022). First, the technician placed the probe on the left lumbar region of the participant in a seated position and drew the renal artery. Subsequently, the technician zoomed the measurement screen and moved the cursor to the renal artery and measured blood flow velocity (BFV) using the pulse Doppler method. The mean blood flow rate was defined as the average of three Doppler waveforms. Next, the technician further zoomed the measurement screen and determined the cross‐sectional area (CSA), and RBF was calculated based on BFV and CSA using the following equation: RBF (mL/min) = BFV (cm/s) × CSA (mm^2^) × 60 (s). Furthermore, we measured peak systolic flow velocity (PSV) and end‐diastolic flow velocity (EDV) using the pulse Doppler method. Renal resistive index (rRI) and renal pulsatility index (rPI), which are indicators of pulsatility and vascular compliance, were calculated as follow (Arbeille et al., 1987; Gosling et al., 1971):

Renal pulsatility index = (peak systolic flow velocity − end‐diastolic flow velocity)/blood flow velocity.

Renal resistive index = (peak systolic flow velocity − end‐diastolic flow velocity)/peak systolic flow velocity.

The assessment of renal hemodynamic was performed in the seated position to remove the effect of posture on renal hemodynamics throughout the experiments. The Doppler angle of incidence in the direction of blood flow was measured to be within 60° to provide an accurate measure of BFV. The technician measures at the origin of the renal artery, allowing that the Doppler probe was measuring the same part of the renal artery at each timepoint. By positioning the probe in advance and marking the location where the probe was to be placed, the same location was used each time. At the time of measurement, the technician asked the participants to hold their breath and placed the probe in the same location. The measurement was performed using a high‐resolution echo. Renal hemodynamic assessments were performed by the same technician throughout the study period. For data reproducibility, we have already reported that the intraclass correlation coefficient of renal hemodynamic assessment with ultrasound echo was 0.96 in previous study (Kawakami et al., 2018). There are several advantages of evaluating renal hemodynamics using ultrasound echo, which can noninvasively visualize blood vessels by applying a probe, thereby diminishing the burden on the participants. Another benefit of ultrasound echo is that it evaluates the BFV and CSA of the renal artery in the kidney independently, enabling the examination of whether the variation in RBF is derived from variations in the BFV and/or CSA.

### 2.6 The determination of blood and urinary biomarkers

Blood and urine samples were collected on the morning after an 8‐h overnight fast, and adrenaline, noradrenaline, aldosterone, serum creatinine (sCr), and cystatin C (sCys) levels were measured. Plasma renin activity (PRA) was also assessed. Additionally, all participants provided urine samples for the examination of creatinine, albumin, N‐acetyl‐beta‐d‐glucosaminidase (NAG), and liver‐type fatty acid‐binding protein (L‐FABP) levels to assess kidney injury in response to exercise. Furthermore, we measured urinary kidney injury molecule 1 (KIM‐1) in duplicate using the sandwich enzyme‐linked immunosorbent assay (ELISA) kit (Human Urinary KIM‐1 Quantikine ELISA Kit, R&D Systems, United States) and urinary neutrophil gelatinase‐associated lipocalin (NGAL) in duplicate using the sandwich ELISA kit (Human NGAL Quantikine ELISA Kit, R&D Systems, United States) to assess kidney injury response to exercise.

Moreover, AKI was defined based on the Acute Kidney Injury Network criteria for AKI severity using either increased sCr or decreased glomerular filtration rate (GFR) (Mehta et al., 2007). Stage 1 was defined as a 1.5–2‐fold or 0.3 mg/dL increase in sCr concentration from baseline to peak value. Stage 2 was defined as a 2–3‐fold increase in sCr concentration. We used the cut‐off values with respect to urinary L‐FABP (uL‐FABP) (8.4 μg/g creatinine) and (micro‐) albuminuria (30 mg/g creatinine) to define kidney injury. Further, we used the cut‐off values for urinary KIM‐1 (uKIM‐1) (31–40 years: 2.14 μg/g creatinine, 41–50 years: 2.24 μg/g creatinine) and urinary NGAL (uNGAL) (31–40 years: 122.1 μg/g creatinine, 41–50 years: 127.6 μg/g creatinine) to define kidney injury or damage (Pennemans et al., 2013).

Each blood sample was centrifuged for 10 min at 1750 × *g* at 4°C, and a part of the urine sample was centrifuged for 5 min at 400 × *g* at 4°C. Samples were stored at −80°C until analysis. The analyses were performed by a commercial blood and urine company (LSI Medience Corp., Tokyo, Japan). Furthermore, the estimated GFR (eGFR) with sCr or sCys and filtration fraction (FF) served as the indicators of renal function and renal hemodynamics and were calculated as described previously (Kawakami et al., 2022).

### 2.7 Statistical analysis

A two‐way linear mixed model was used to examine any differences in the dependent variables over time (before exercise, immediately after exercise, and 30 and 60 min after exercise) and between exercise conditions (HIIE or MICE) with random effects to control for participants in the repeated‐measures design. Bonferroni's post hoc test was performed for significant main or interaction effects. Furthermore, we determined the association between eGFR and the changes in RBF, rPI, or rRI from pre to post 0 (post 0), post 30 (post 30), and post 60 (post 60) using the simple linear regression analysis. All statistical analyses were performed using Prism version 9.5.1 (GraphPad Software, San Diego, CA, USA). Differences were considered statistically significant at *p* < 0.05. Data are presented as the means ± standard deviations.

## 3 RESULTS

### 3.1 Participant characteristics

Table 1 represents the participant characteristics in this study. We included middle‐aged individuals (37 ± 8 years) with relatively well‐maintained renal function (eGFR 82 ± 9 mL/min/1.73 m^2^) in this study.

**TABLE 1:** Participant characteristics.

Age, year	37 ± 8
Height, m	1.72 ± 0.05
Weight, kg	67.4 ± 6.0
Body Mass Index, kg/m^2^	22.9 ± 2.0
Systolic blood pressure, mmHg	110 ± 7
Diastolic blood pressure, mmHg	74 ± 9
Serum creatinine, mg/dL	0.86 ± 0.07
eGFR, mL/min/1.73 m^2^	82 ± 9
VO_2_peak, mL/kg/min	37.0 ± 9.9
VO_2_@LT, mL/kg/min	13.2 ± 4.6
VO_2_@OBLA, mL/kg/min	21.7 ± 6.5

*Note*: Data are expressed as the mean ± SD.

Abbreviations: eGFR, estimated glomerular filtration rate; LT, lactate threshold; OBLA, onset of blood lactate accumulation; VO2peak, peak oxygen uptake.

### 3.2 Renal hemodynamics following HIIE and MICE conditions

**FIGURE 3:**
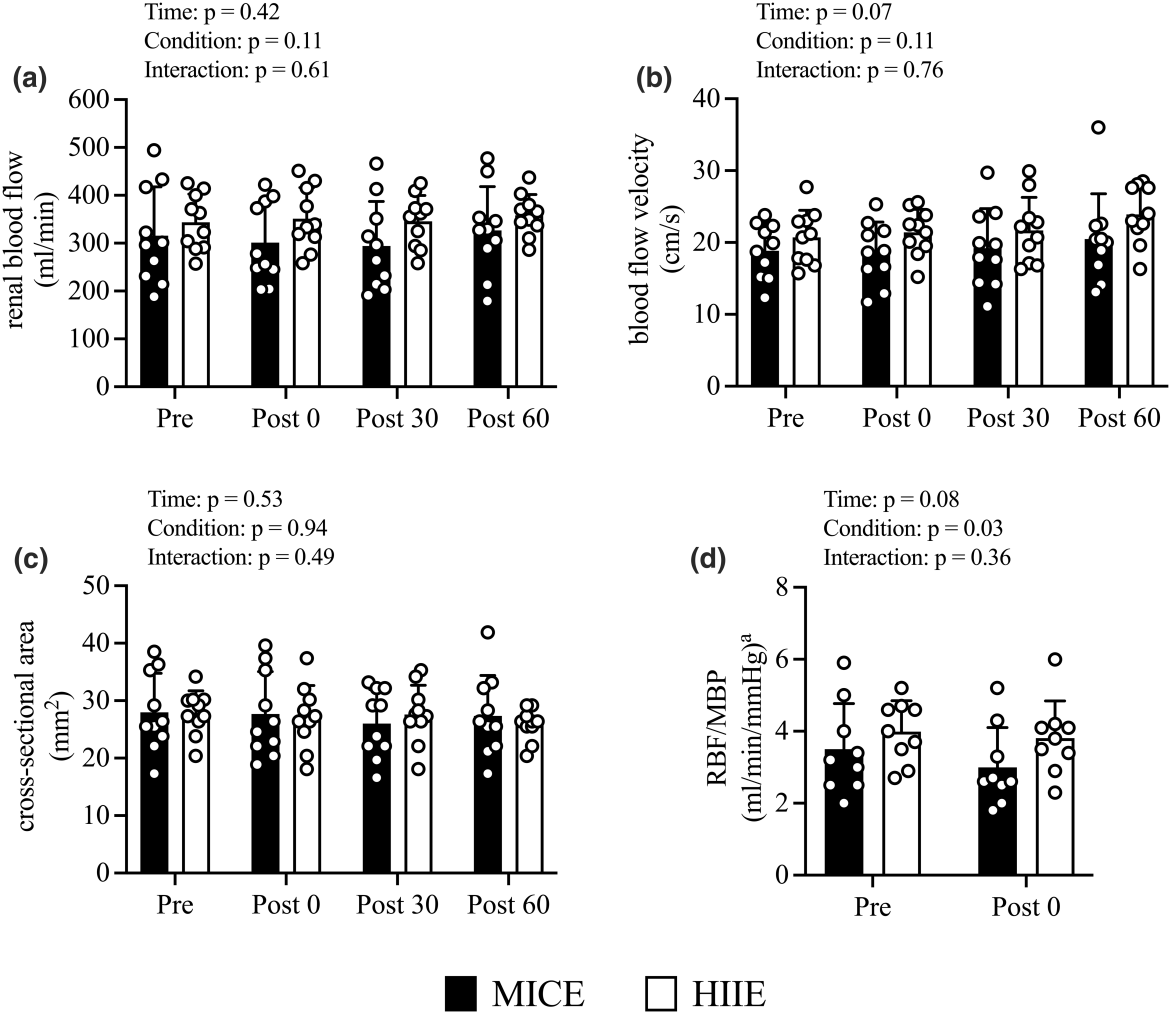
Changes in renal blood flow (a), blood flow velocity (b), cross‐sectional area (c) and renal blood flow corrected for mean blood pressure (d) from before exercise (*Pre*) to immediately after exercise (*Post 0*) to 30 min post‐exercise (*Post 30*) to 60 min post‐exercise (*Post 60*) in MICE (filled bar, *n* = 10) and HIIE (open bar, *n* = 10). Data are the mean ± standard deviation. ^a^Data are available as follows: MICE, *n* = 9; HIIE, *n* = 9. Open circles represent individual data. Multiple pairwise comparisons were corrected using the Bonferroni method.

Figure 3 shows the changes in renal hemodynamics following HIIE and MICE. There were no condition‐by‐time interaction, condition, or time effects regarding RBF (Figure 3a). There was no condition‐by‐time interaction effect regarding changes in BFV (Figure 3b) and CSA (Figure 3c). Moreover, there were no time and condition effects regarding BFV and CSA. There were no condition‐by‐time interaction or time effects; however, there was a condition effect (*p* = 0.03) regarding RBF corrected for mean blood pressure (Figure 3d).

### 3.3 Renal function and injury

**FIGURE 4:**
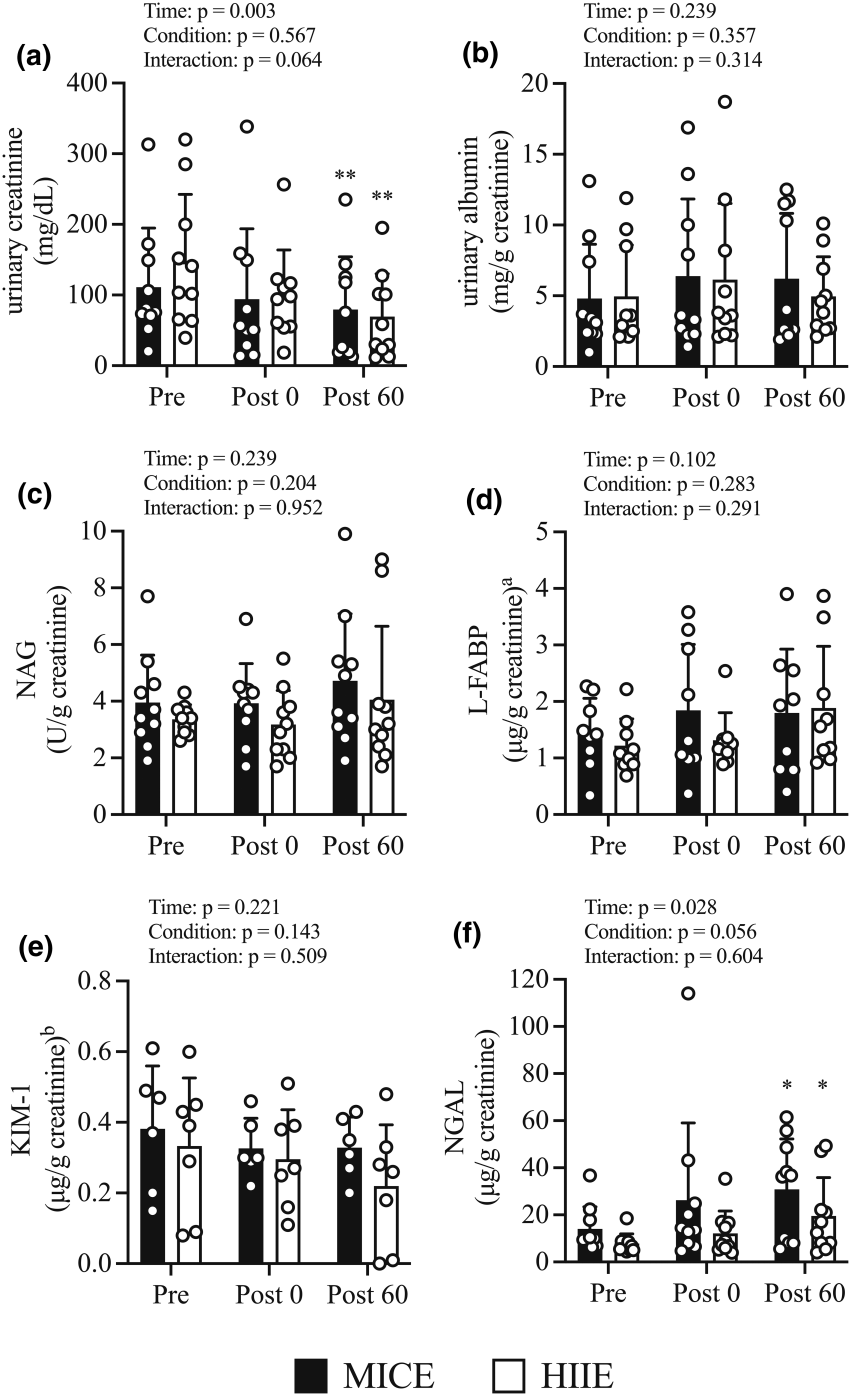
Variations in acute kidney injury biomarkers before and after exercise and recovery period. Changes in urinary creatinine (a), urinary albumin (b), N‐acetyl‐beta‐d‐glucosaminidase (c), liver‐type fatty acid‐binding protein (d), kidney molecule injury 1 (e), neutrophil gelatinase‐associated lipocalin (f) from before exercise (*Pre*) to immediately after exercise (*Post 0*) to 60 min post‐exercise (*Post 60*) in MICE (filled bar, *n* = 10) and HIIE (open bar, *n* = 10). Data are the mean ± standard deviation. *p < 0.05, **p < 0.01 versus *Pre*. ^a^Data are available as follows: MICE, *n* = 9; HIIE, *n* = 9. ^b^Data are available as follows: MICE, *n* = 6; HIIE, *n* = 7. Open circles represent individual data. Multiple pairwise comparisons were corrected using the Bonferroni method.

Figure 4 shows the urinary creatinine (uCr), urinary albumin (uAlb), urinary NAG (uNAG), uL‐FABP, uKIM‐1, and uNGAL excretion responses to HIIE or MICE. All AKI biomarkers were corrected for uCr levels. There were no condition‐by‐time interaction or condition effects; however, there was a time effect (*p* = 0.003) regarding uCr levels (Figure 4a). The uCr levels showed no significant changes at *Post 0* compared to *Pre* but showed a significant decrease at *Post 60* (*p* = 0.002) in both conditions.

There were no condition‐by‐time interaction, condition, or time effect regarding changes in uAlb (Figure 4b), uNAG (Figure 4c), uL‐FABP (Figure 4d), and uKIM‐1 (Figure 4e) levels. There were no condition‐by‐time interaction or condition effects; however, there was a time effect regarding uNGAL levels (Figure 4f; *p* = 0.028). The uNGAL levels showed no significant changes at *Post 0* compared to *Pre* and a significant increase at *Post 60* (*p* = 0.017) in both conditions.

**FIGURE 5:**
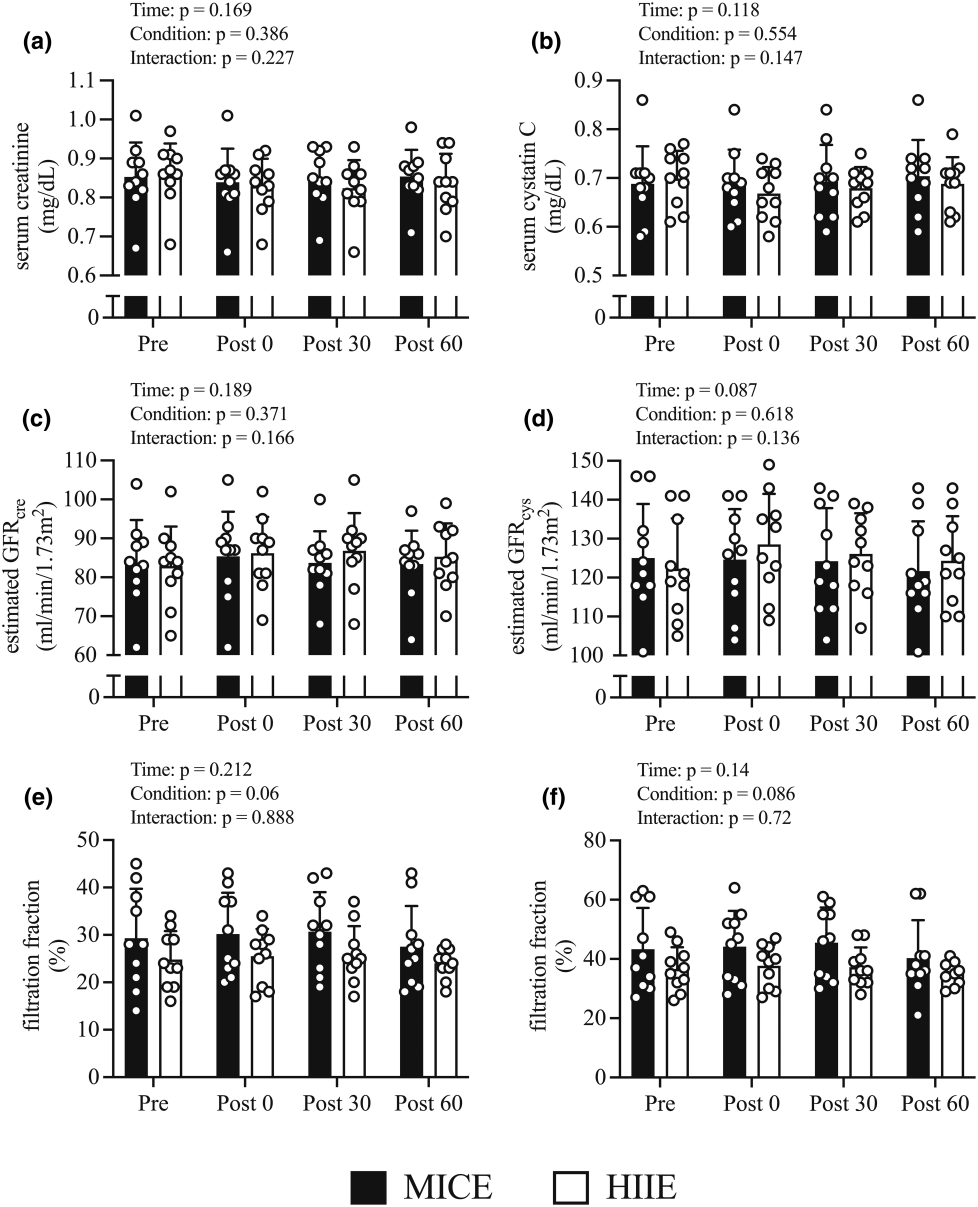
Variations in renal function parameters before and after exercise and recovery period. Changes in serum creatinine (a), serum cystatin C (b), estimated GFR_cre_ (c), estimated GFR_cys_ (d), filtration fraction (creatinine) (e), filtration fraction (cystatin C) (f) from before exercise (*Pre*) to immediately after exercise (*Post 0*) to 30 min post‐exercise (*Post 30*) to 60 min post‐exercise (*Post 60*) in MICE (filled bar, *n* = 10) and HIIE (open bar, *n* = 10). Data are the mean ± standard deviation. *GFR* glomerular filtration rate. Open circles represent individual data. Multiple pairwise comparisons were corrected using the Bonferroni method.

The changes in renal function parameters before and immediately after exercise and during the recovery period are shown in Figure 5. There were no condition‐by‐time interaction, condition, or time effects regarding changes in sCr (Figure 5a), sCys (Figure 5b), eGFR_cre_ (Figure 5c), eGFR_cys_ (Figure 5d), FF_cre_ (Figure 5e), and FF_cys_ (Figure 5f) levels.

### 3.4 Other renal hemodynamic parameters following HIIE and MICE conditions

**FIGURE 6:**
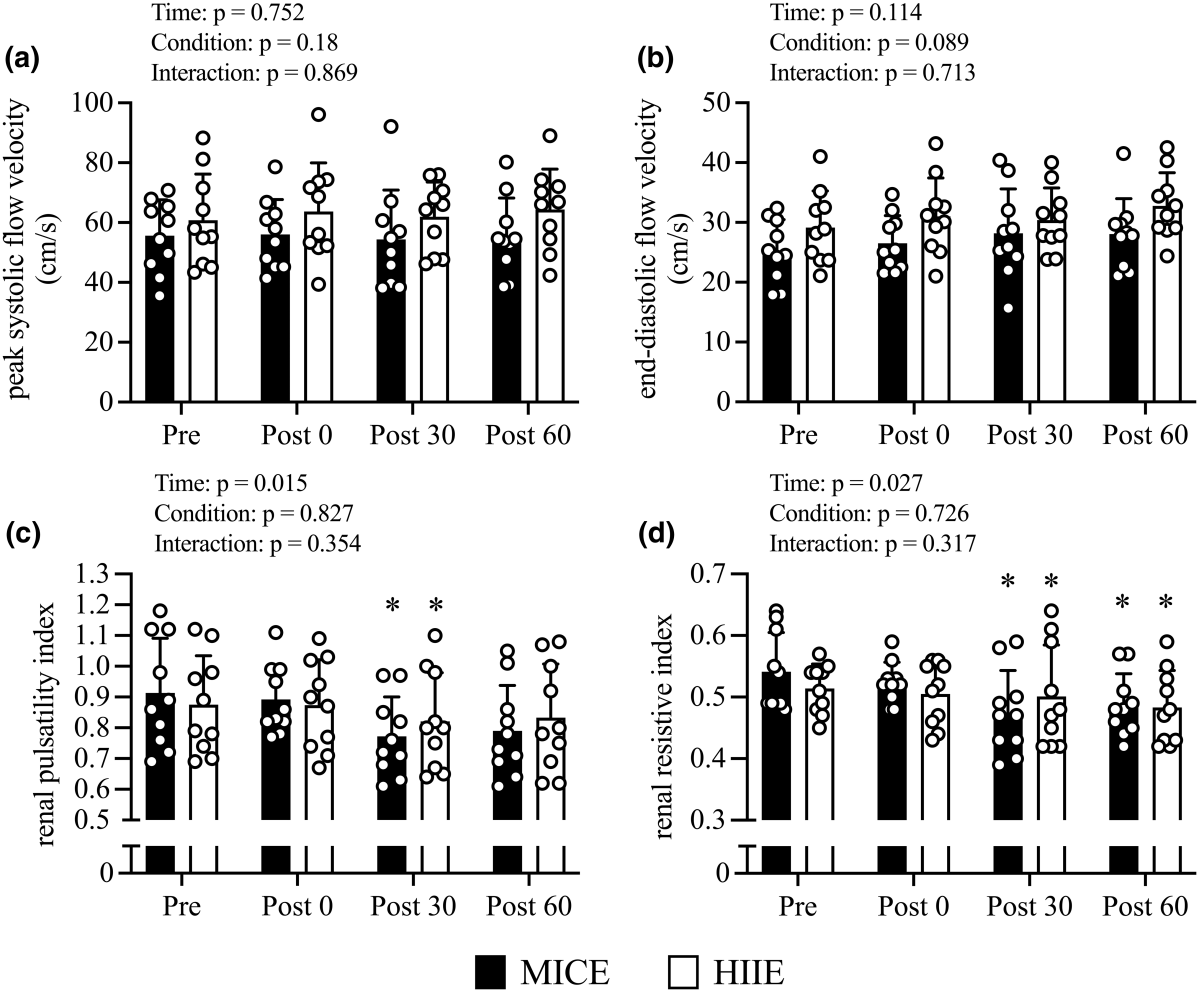
Changes in peak systolic flow velocity (a), end‐diastolic flow velocity (b), renal pulsatility index (c), renal resistive index (d) from before exercise (*Pre*) to immediately after exercise (*Post 0*) to 30 min post‐exercise (*Post 30*) to 60 min post‐exercise (*Post 60*) in MICE (filled bar, *n* = 10) and HIIE (open bar, *n* = 10). Data are the mean ± standard deviation. *Renal pulsatility index* (peak systolic flow velocity—end‐diastolic flow velocity)/blood flow velocity, *Renal resistive index* (peak systolic flow velocity—end‐diastolic flow velocity)/peak systolic flow velocity. *p < 0.05 versus *Pre*. Open circles represent individual data. Multiple pairwise comparisons were corrected using the Bonferroni method.

Figure 6 shows the variations in other renal hemodynamic parameters before and immediately after exercise and during the recovery period under both conditions. There were no condition‐by‐time interaction, condition, or time effect regarding changes in PSV (Figure 6a) and EDV (Figure 6b). There was no condition‐by‐time interaction effect regarding rPI (Figure 6c) and rRI (Figure 6d); however, there was a significant time effect (rPI, *p* = 0.015; rRI, *p* = 0.027). The rPI showed no significant changes at *Post 0* compared to *Pre* and showed a significant decrease at *Post 30* (*p* = 0.025) in both conditions. Moreover, the rRI showed no significant changes in *Post 0* compared to *Pre* and showed a significant decrease at *Post 30* (*p* = 0.048) and *Post 60* (*p* = 0.03) in both conditions.

### 3.5 Biochemical parameters associated with the regulation of renal hemodynamics

**FIGURE 7:**
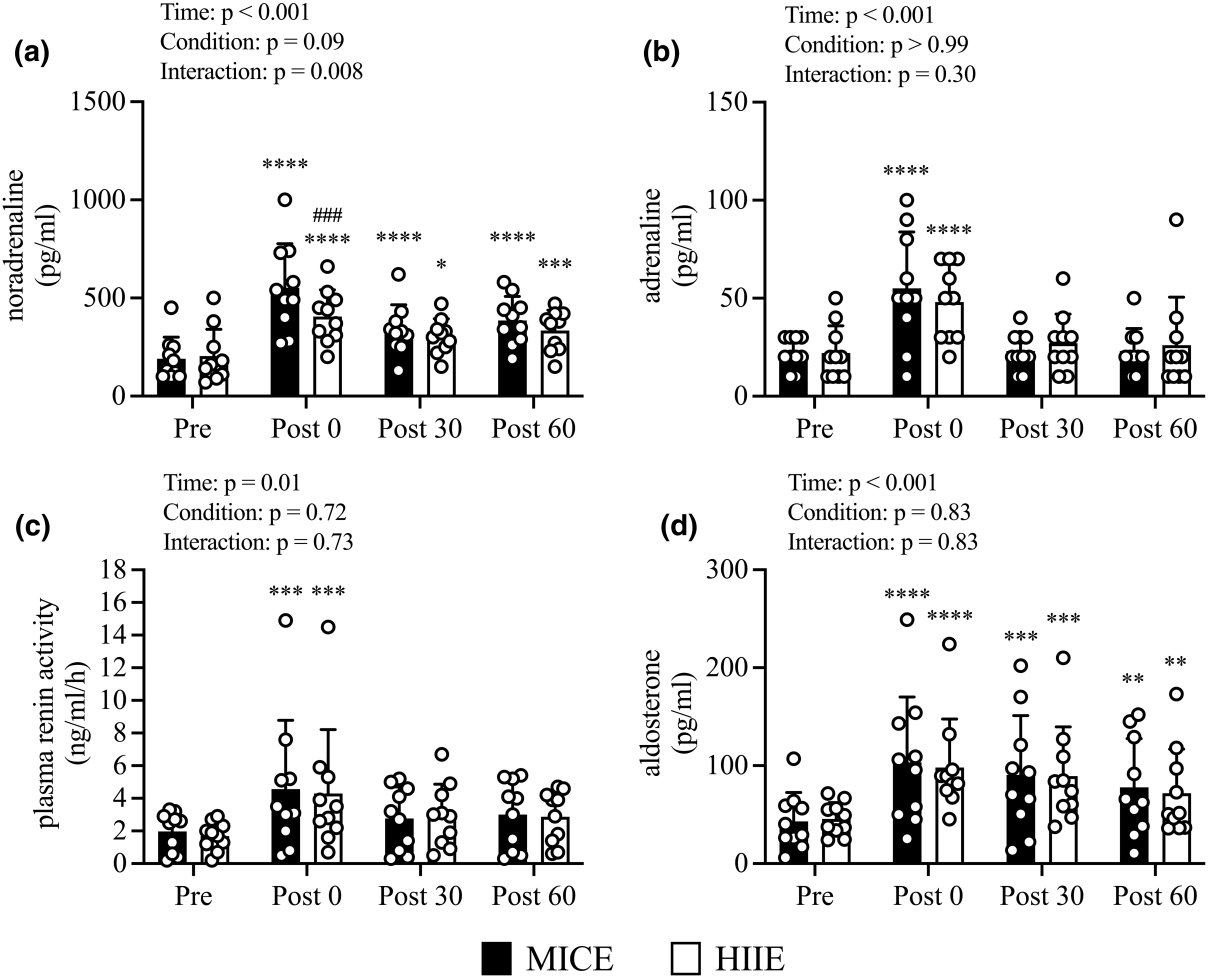
Changes in noradrenaline (a), adrenaline (b), plasma renin activity (c), aldosterone (d) from before exercise (*Pre*) to immediately after exercise (*Post 0*) to 30 min post‐exercise (*Post 30*) to 60 min post‐exercise (*Post 60*) in MICE (filled bar, *n* = 10) and HIIE (open bar, *n* = 10). Data are the mean ± standard deviation. *p < 0.05, **p < 0.01, ***p < 0.005, ****p < 0.001 versus *Pre*. ♯*p* < 0.05, ♯♯*p* < 0.01, ♯♯♯*p* < 0.05 versus MICE. Open circles represent individual data. Multiple pairwise comparisons were corrected using the Bonferroni method.

A condition‐by‐time interaction effect was observed regarding noradrenaline levels (Figure 7a; *p* = 0.008). Noradrenaline levels at *Pre* did not differ between conditions; however, the noradrenaline level at *Post 0* was lower in the HIIE conditions than in the MICE conditions (*p* = 0.003). In the HIIE condition, noradrenaline levels showed a significant increase at *Post 0* compared to *Pre* (*p* < 0.001) and remained significantly higher at *Post 30* (*p* = 0.031) and *Post 60* (*p* = 0.004). In contrast, noradrenaline levels showed a significant increase at *Post 0* compared to *Pre* (*p* < 0.001) and remained significantly higher at *Post 30* (*p* < 0.001) and *Post 60* (*p* < 0.001) in the MICE condition.

There was no condition‐by‐time interaction effect regarding the changes in adrenaline (Figure 7b), PRA (Figure 7c), and aldosterone (Figure 7d) levels. However, there were time effects regarding adrenaline (*p* < 0.001), PRA (*p* = 0.01), and aldosterone (*p* < 0.001) levels. Compared to *Pre*, adrenaline (*p* < 0.001) and PRA (*p* = 0.003) levels increased significantly at *Post 0* and showed no significant changes at *Post 30* or *Post 60* in both conditions. Aldosterone levels showed a significant increase at *Post 0* compared to *Pre* (*p* < 0.001) and remained significantly higher at *Post 30* (*p* = 0.001) and *Post 60* (*p* = 0.009) in both conditions.

### 3.6 Physiological parameters following HIIE and MICE conditions

Table 2 shows the variations in physiological parameters before and immediately after exercise and during the recovery period under both conditions. A condition‐by‐time interaction effect was observed regarding HR (*p* < 0.001). HR at *Pre* did not differ between conditions and HR showed a significant increase at *Post 0* compared to *Pre* in both conditions (*p* < 0.001, *p* < 0.001, respectively). However, HR at *Post 0* was higher in the HIIE conditions than in the MICE conditions (*p* < 0.001). There was no condition‐by‐time interaction or condition effects; however, there was a time effect (*p* < 0.001) regarding systolic blood pressure. There were no condition‐by‐time interaction, condition, or time effect regarding changes in diastolic blood pressure. A condition‐by‐time interaction effect was observed regarding blood lactate concentrations (*p* = 0.011). In the HIIE condition, blood lactate concentrations showed a significant increase after exercise (*p* = 0.004). In contrast, blood lactate concentrations showed no significant changes after exercise in the MICE condition. Moreover, blood lactate concentration after exercise was higher in the HIIE conditions than in the MICE conditions (*p* = 0.011).

**TABLE 2:** Comparison of physiological parameters before and after HIIE and MICE, and recovery phase.

	HIIE (*n* = 10)		MICE (*n* = 10)		Interaction effect	Time effect	Condition effect
	Before ex	After ex	Before ex	After ex			
Heart rate, bpm	66 ± 12	115 ± 16^** ♯♯^	67 ± 10	100 ± 14**	*p* < 0.001	*p* < 0.001	*p* = 0.001
Systolic blood pressure, mmHg	108 ± 9	144 ± 12	107 ± 10	146 ± 15	*p* = 0.181	*p* < 0.001	p = 0.874
Diastolic blood pressure, mmHg	75 ± 9	70 ± 11	76 ± 10	76 ± 13	*p* = 0.382	*p* = 0.25	*p* = 0.183
Mean blood pressure, mmHg	86 ± 8	94 ± 10	87 ± 9	99 ± 13	*p* = 0.266	*p* = 0.004	*p* = 0.333
Blood lactate concentration, mmol/L	1.6 ± 0.2	2.8 ±0.4^** ♯^	1.5 ± 0.3	1.9 ± 0.4	*p* = 0.011	*p* = 0.004	*p* = 0.013
Energy expenditure per session, kcal	139 ± 43		139 ± 43				
Total duration of the session, min	32 ± 3		30 ± 0				
Work load, watts	115 ± 35		63 ± 27				
Heart rate reserve, %	47 ± 13		31 ± 13				

*Note*: Data are expressed as the mean SD.

Abbreviations: HIIE, high‐intensity intermittent exercise; MICE, moderate‐intensity continuous exercise. *p < 0.05, **p < 0.01 versus before Ex. ♯*p* < 0.05, ♯♯*p* < 0.01 versus MICE.

### 3.7 The association between eGFR and the changes in RBF, rPI, or rRI

The eGFR_cys_ was negatively associated with ∆rPI_post 30 (*p* = 0.011) and ∆rRI_post 30 (*p* = 0.004). However, there were no significant association between eGFR_cys_ and other parameters. eGFR_cre_ showed positive association with ∆RBF_post 60 (*p* = 0.018), but it did not show an obvious association with other parameters (Table 3).

**TABLE 3:** Simple correlations between eGFR and changes in renal hemodynamic parameters.

	eGFR_cys_		eGFR_cre_	
	*r*	*p*	*r*	*p*
∆RBF_post 0	−0.133	0.576	0.349	0.131
∆RBF_post 30	0.304	0.193	0.424	0.062
∆RBF_post 60	0.014	0.965	0.524	0.018
∆rPI_post 0	−0.323	0.164	0.006	0.981
∆rPI_post 30	−0.558	0.011	−0.159	0.503
∆rPI_post 60	−0.052	0.827	−0.131	0.582
∆rRI_post 0	−0.358	0.121	0.348	0.133
∆rRI_post 30	−0.611	0.004	−0.191	0.420
∆rRI_post 60	−0.069	0.774	0.101	0.673

Abbreviations: eGFR_cre_, estimated glomerular filtration with serum creatinine; eGFR_cys_, estimated glomerular filtration with serum cystatin C; RBF, renal blood flow; rPI, renal pulsatility index; rRI, renal resistive index.

## 4 DISCUSSION

The current study is the first to investigate the effects of HIIE and MICE on renal hemodynamics, including RBF, using ultrasound echo in middle‐aged individuals. Furthermore, we investigated the effects of HIIE and MICE on renal function parameters, and AKI biomarkers. The main finding was that even under HIIE conditions, the RBF, eGFR levels, and FF were maintained, and there were no changes in AKI biomarkers. Additionally, HIIE was found to have an effect on renal hemodynamics, renal function, and AKI biomarkers similar to that of MICE.

Recently, HIIE was noted as an exercise condition that can improve cardiorespiratory fitness and decrease CVD risk factors in both healthy and diseased populations (Ramos et al., 2015; Taylor et al., 2020; Weston et al., 2014). In addition, HIIE has been shown to be effective in enhancing mitochondrial biogenesis in skeletal muscles (Tjønna et al., 2008; Wisløff et al., 2007). A recent clinical pilot study demonstrated that HIIE might be a feasible and safe option for patients with CKD and that HIIE and MICE have similar benefits on exercise capacity and skeletal muscle protein responses (Beetham et al., 2019). Therefore, HIIE, which has greater health‐enhancing benefits, may be an attractive option for patients with CKD who are more likely to lose their cardiorespiratory fitness and skeletal muscle mass. However, it is well known that high‐intensity exercise affects renal hemodynamics and that the decrease in RBF depends on the intensity of the exercise (Grimby, 1965; Kawakami et al., 2018; Kotoku et al., 2019). To prevent a decrease in renal function, we considered whether HIIE could be a safe and effective exercise option that does not impair the kidney function.

In this study, we examined how HIIE affected renal hemodynamics using ultrasound echo and demonstrated that HIIE did not induce a significant decrease in RBF, suggesting that high‐intensity exercise can be performed without a decrease in RBF when conducted intermittently for short periods. Our previous studies have shown that changes in RBF correspond to changes in CSA and that sympathetic nervous activity may influence these changes (Kawakami et al., 2018; Kotoku et al., 2019). Therefore, we examined the effects of HIIE on BFV and CSA, which constitute RBF, to further explore the effect of exercise on RBF in detail. Our detailed analysis revealed that HIIE has no effect on the BFV or CSA. Furthermore, we observed that HIIE and MICE significantly increased noradrenaline concentrations, which have a strong vasoconstrictive effect; however, the noradrenaline concentrations immediately after exercise were significantly lower under HIIE conditions than under MICE conditions. These results suggest that high‐intensity but intermittent exercise does not decrease the CSA by suppressing the increase in noradrenaline concentration and, consequently, does not decrease RBF.

In general, albuminuria and eGFR are known to be independently associated with AKI (Grams et al., 2010). However, novel biomarkers of kidney injury have recently been shown to be more sensitive than albuminuria and eGFR (Belcher et al., 2011; Siew et al., 2011). These AKI biomarkers may be better suited for the sensitive detection of exercise‐induced kidney injury. In this study, we observed that uAlb and eGFR levels showed no significant changes under HIIE and MICE conditions. Moreover, our results demonstrated that no changes in the uNAG, uL‐FABP, and uKIM‐1 levels were observed under both HIIE and MICE conditions. In contrast, uNGAL concentration exhibited no significant change immediately after exercise and significantly increased 60 min after exercise in both conditions. One possible explanation for the increased NGAL levels with exercise is that aldosterone increases NGAL levels via the activation of mineralocorticoid receptors (MRs). Aldosterone can activate MRs and increase NGAL production by neutrophils, which is mediated by MR, phosphoinositide 3‐kinase, p38, and extracellular signal‐regulated kinase 1/2 pathways (Gilet et al., 2015). We confirmed that aldosterone concentrations were elevated after exercise and remained elevated 60 min after exercise under both conditions, suggesting that elevated aldosterone may be involved in the elevated NGAL levels in this study. Further, uNGAL levels seem to be frequently increased but rarely exceeds normal values when normalized to creatinine (Wołyniec et al., 2020). None exceeded the cut‐off values (31–40 years: 122.1 μg/g creatinine, 41–50 years: 127.6 μg/g creatinine) for defining kidney injury (Pennemans et al., 2013) in this study. Moreover, an increase in uNGAL levels does not necessarily indicate an increase in distal nephron injury because a decrease in the proximal tubular reabsorption of NGAL may also contribute to an increase in uNGAL (Kashani et al., 2017). Taken together, these results suggest that HIIE and MICE conditions have comparable effects on AKI biomarkers, and HIIE does not induce glomerular or proximal tubular injury.

One potential factor contributing to increased AKI biomarkers with exercise is the exercise intensity or duration. This is because increased sympathetic nervous system activity (Grisk, 2020), and dehydration (Chapman et al., 2020) under higher intensity or prolonged exercise have been shown to increase AKI biomarkers via a decrease in RBF. A previous study reported the effects of acute versus prolonged exercise on the levels of eGFR and kidney injury biomarkers in healthy male adults (Bongers et al., 2018). Acute exercise had no effect on the levels of eGFR and AKI biomarkers, whereas prolonged exercise with dehydration was associated with reduced eGFR levels and increased AKI biomarker levels (Bongers et al., 2018). Another study demonstrated changes in uKIM‐1 and uNGAL levels following intensive exercise, indicating that intensive exercise induces a significant increase in uKIM‐1 levels and a non‐significant change in uNGAL levels (Wołyniec et al., 2018). Moreover, a recent study examined the effect of HIIE on AKI biomarkers, demonstrating that HIIE with hypohydration exacerbates the levels of AKI biomarkers and suggesting that kidney injury by HIIE may be mediated by a reduction in RBF (Juett et al., 2021). Collectively, these results suggest that HIIE without decreased RBF does not induce glomerular or tubular injury.

The kidney is a mass of peripheral blood vessels, and although the importance of determining the effects of exercise on renal microcirculation is highly significant, much remains unknown. An important indicator of the relationship between renal hemodynamics and renal function is the FF, which is eGFR divided by RBF. In this study, the RBF and eGFR levels did not significantly decrease with exercise, and there was no significant change in the FF under either HIIE or MICE conditions. The increase in FF is likely one of the causes of proteinuria in subjects that undergo exercise. Moreover, exercise‐induced proteinuria levels may not have increased because the FF showed no change in this study. Also, increased lactate and acidosis may alter glomerular permeability and inhibit the tubular transportation of small molecular weight proteins. Previous study has reported that marked albuminuria is observed with marked increase in blood lactate concentrations (Poortmans & Vanderstraeten, 1994). In our study, the blood lactate concentrations following HIIE were higher than those following MICE (MICE: 1.9 ± 0.4 mmol/L, HIIE: 2.8 ± 0.4 mmol/L), but not as elevated (pre‐HIIE: 1.6 ± 0.2 mmol/L, post‐HIIE: 2.8 ± 0.4 mmol/L). Previous study has reported that intermittent exercise induce a less increase in blood lactate concentrations compared to continuous exercise (Meri̇c et al., 2022), suggesting intermittent exercise may be less likely to increase albuminuria. Therefore, our findings suggest that no significant increase in urinary albumin was observed following HIIE in this study. Additionally, rRI (Arbeille et al., 1987) and rPI (Gosling et al., 1971), which are measures of intrarenal hemodynamics derived from ultrasound echo, can be useful tools for determining detailed intrarenal hemodynamic responses to exercise. We observed that rRI and rPI decreased 30 min after the exercise, and a significant decrease in rRI persisted for 60 min after exercise under both conditions. Our recently published works found that decreases in rRI and rPI were observed 30 min after MICE (Kawakami et al., 2022) and 60 min after incremental maximal exercise (Kawakami et al., 2023). Decreases in rRI and rPI after exercise indicate reduced renal peripheral vascular resistance, and exercise can have a beneficial effect on the renal microcirculation. However, further detailed studies are needed to elucidate the influence of exercise on renal microcirculation.

This study had several limitations. Firstly, one of the main limitations of this study is the relatively small sample size, which should be considered when interpreting the results. Secondly, Renal hemodynamic assessments were conducted immediately after exercise in this study. As the kidneys move with respiration and body movement, breathing needs to pause when assessing renal hemodynamics, this makes it difficult to accurately assess renal hemodynamics during exercise. Therefore, we asked participants to cease all body movement as much as possible immediately after exercise and promptly assess renal hemodynamics using high‐resolution ultrasound echo, and we have evaluated the influences of HIIE or MICE on renal hemodynamics in this study. In the future, a better understanding of the influence of exercise on renal hemodynamics may be obtained if renal hemodynamic assessment can be performed during exercise. Thirdly, we included middle‐aged males (37 ± 8 years) with relatively preserved renal function (eGFR ≥60 mL/min/1.73 m^2^) in this study. The renal hemodynamic response to exercise may vary depending on the severity of renal functional impairment. Therefore, similar studies using non‐dialysis CKD patients (eGFR <60 mL/min/1.73 m^2^) should be performed in the future for clinical application. Finally, we did not measure urine cystatin C, osmolality and flow rate in this study. The assessment of urinary AKI biomarkers is likely to be influenced by changes in hydration status. There has been examined the urinary biomarkers corrected for urinary creatinine, cystatin C and osmolality for changes in hydration status (Bongers et al., 2017, 2018). It is important to correct urinary biomarkers for changes in hydration status and urine flow rate to estimate the effect of exercise on kidney injury. However, these correction methods both have limitations in dynamic state such as exercise and correction of urinary biomarker remains problematic (Junglee et al., 2012). Therefore, further study is needed to examine the changes in urinary biomarkers corrected for urine cystatin C, osmolality and flow rate for changes in hydration status and biomarker excretion rate.

The health challenges become more diverse as the global population ages. Especially, one in eight adults has CKD, and threat of CKD is increasing worldwide (Kovesdy, 2022). CKD is a strong risk factor for the development of cardiovascular disease and the all‐cause mortality (Go et al., 2004; Matsushita et al., 2022), the main treatment modalities for CKD patients are nutritional and drug therapy. Exercise therapy may be an effective nonpharmacologic strategy (Beetham et al., 2022; Howden et al., 2015; Weiner et al., 2023), therefore, it is important to properly understand the influence of exercise on the kidneys to enable effective exercise programs to be designed to prevent the renal function decline. We utilized ultrasound echo to understand the influence of HIIE on renal hemodynamics and provided novel evidence that high‐intensity exercise can be performed without decreased RBF or increased kidney injury risk when conducted intermittently for short periods. Our future research plans are to elucidate the exercise conditions that are beneficial and harmless to the kidneys, and it is significant. The use of ultrasound echo allowed detailed information on the intrarenal hemodynamic response to exercise to be obtained, while minimizing the burden on the subjects. Therefore, non‐invasive observation of renal hemodynamic changes following exercise using ultrasound echo may be clinically useful.

In conclusion, we investigated the effect of HIIE on renal hemodynamics using ultrasound echo in middle‐aged individuals. Our findings demonstrate that high‐intensity exercise can be performed without a decrease in RBF when performed intermittently for short periods. It also demonstrates that HIIE does not alter urinary liver‐type fatty acid‐binding protein and kidney injury molecule‐1. Collectively, our data suggest that HIIE without decreased RBF does not induce glomerular or tubular injury.

## AUTHOR CONTRIBUTIONS

S. Kawakami, T.Y., and R.M. conceived and designed research; S. Kawakami, T.Y., S.K., and A.I. performed experiments; S Kawakami, T.Y., and K.F. gathered the participants; S. Kawakami analyzed data; S. Kawakami, T.Y., and R.M. interpreted results of experiments; S. Kawakami prepared figures; S. Kawakami drafted manuscript; S. Kawakami, T.Y., S.K., A.I., K.F., T.M., S.N., K.M., Y.U., Y.H., and R.M. edited and revised manuscript; and all authors approved final version of manuscript.

## FUNDING INFORMATION

This work was supported by a Grant‐in‐Aid for Scientific Research (C) from the Japan Society for the Promotion of Science (JSPS) (to SK, no. 22K11489), and Fukuoka University Institute for Physical Activity, Fukuoka, Japan.

## CONFLICT OF INTEREST STATEMENT

No conflicts of interest, financial or otherwise, are declared by the authors.

## ETHICS STATEMENT

All procedures involving human participants were performed in accordance with the ethical standards of the institutional and/or national research committee at which the studies were conducted (Ethics Committee of Fukuoka University Approval No. 22‐06‐M1).

## Data Availability

The data that support the findings of the present study are available from the corresponding author upon reasonable request.
